# Effects of Etanercept and Adalimumab on Serum Levels of Cartilage Remodeling Markers in Women with Rheumatoid Arthritis

**DOI:** 10.3390/jcm12165185

**Published:** 2023-08-09

**Authors:** Anna Szeremeta, Agnieszka Jura-Półtorak, Aleksandra Zoń-Giebel, Krystyna Olczyk, Katarzyna Komosińska-Vassev

**Affiliations:** 1 Department of Clinical Chemistry and Laboratory Diagnostics, Faculty of Pharmaceutical Sciences in Sosnowiec, Medical University of Silesia, Katowice, Jedności 8, 41-200 Sosnowiec, Poland; ajura@sum.edu.pl (A.J.-P.); olczyk@sum.edu.pl (K.O.); kvassev@sum.edu.pl (K.K.-V.); 2 Department of Rheumatology and Rehabilitation, Specialty Hospital No. 1, Żeromskiego 7, 41-902 Bytom, Poland; azongiebel@gmail.com

**Keywords:** rheumatoid arthritis, etanercept, adalimumab, cartilage remodeling markers, C2C, PIICP, COMP, MMP-3

## Abstract

Tumor necrosis factor α inhibitor (TNFαI) therapy is associated with a significant inhibition of radiographic progression, resulting in improved physical function and quality of life among patients with rheumatoid arthritis (RA). The mechanism by which TNFαI prevent joint destruction is still unknown. In this study, the effect of 15-month anti-TNF-α therapy in combination with methotrexate on circulating levels of biochemical markers of cartilage turnover in female RA patients was assessed. Serum levels of collagen type II C-terminal cleavage neoepitope (C2C), C-terminal propeptide of type II collagen (PIICP), cartilage oligomeric matrix protein (COMP), and matrix metalloproteinase-3 (MMP-3) were evaluated using immunoassays at baseline and 15 months after the start of TNFαI treatment. Baseline COMP, C2C, and MMP-3 levels and C2C/PIICP ratios were significantly higher in women with RA compared with those observed in the healthy subjects. No differences in PIICP levels between the controls and the women with RA were observed. After 15 months of TNFαI treatment, serum levels of C2C, COMP, and MMP-3 decreased, whereas the levels of PIICP increased but were still not different from those of the controls. These changes were accompanied by significantly reduced C2C/PIICP ratios. Before the start of TNFαI therapy, serum levels of COMP significantly correlated with the patients’ ages (*p* < 0.05) and their 28-joint disease activity score values based on their erythrocyte sedimentation rates (DAS28-ESR; *p* < 0.05). Moreover, multiple linear regression analysis showed that baseline COMP levels retained a significant association with DAS28-ESR value (β = 287.74, *p* = 0.022, R^2^ model = 0.25) after model adjustments. The largest area under the ROC curve was obtained for C2C/PIICP ratios (AUC: 0.830, 95% CI: 0.727–0.932, *p* < 0.001). Our results suggest that long-term anti-TNF-α therapy combined with MTX has a beneficial effect on cartilage remodeling that is associated with clinical improvement among RA patients. Serum C2C/PIICP ratios may help to monitor the effectiveness of anti-TNF-α treatment among RA patients.

## 1. Introduction

Rheumatoid arthritis (RA) is the most common form of autoimmune arthritis. It is characterized by progressive cartilage damage and osseous erosion, significant pain, and joint dysfunction, which are the main causes of long-term disability. RA affects millions of individuals worldwide, increases in prevalence with age, and affects more women than men [1,2].

**Figure 1:**
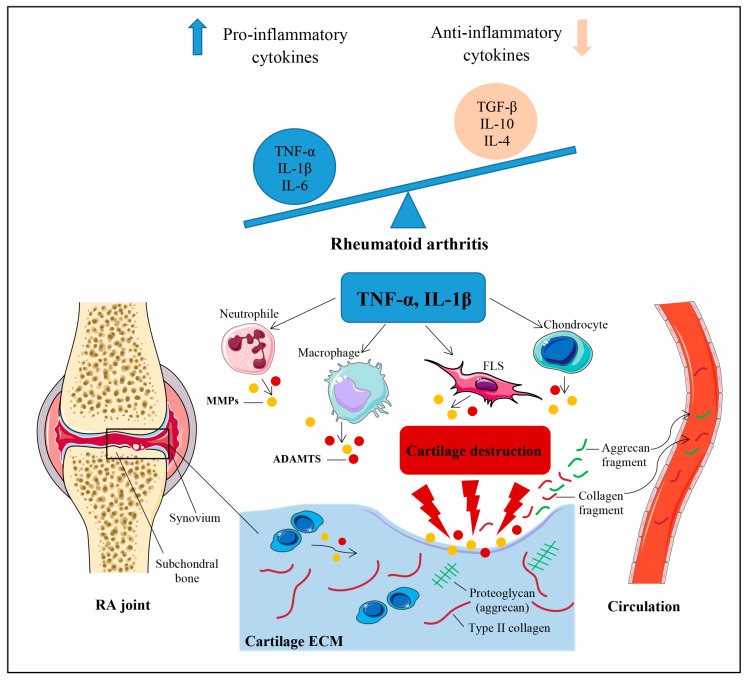
Schematic representation of mechanisms leading to the initiation and progression of cartilage breakdown in RA. Cartilage destruction in rheumatoid arthritis (RA) essentially results from an imbalance between pro- and anti-inflammatory cytokine activities. The key pro-inflammatory cytokines pertaining to RA are TNF-α and IL-1β. These cytokines stimulate chondrocytes, synovial fibroblasts (FLS), macrophages, and neutrophils to produce matrix metalloproteinases (MMPs) and various types of disintegrin and metalloproteinase with thrombospondin motifs (ADAMTS) that play a critical role in the proteolytic cleavage of the cartilage extracellular matrix (ECM) components. Collagenous and non-collagenous fragments of ECM components are released within the joint cavity, where they may be filtered into the circulation and urine. IL, interleukin; TGF-β, transforming growth factor β; TNF-α, tumor necrosis factor α.

Although the mechanisms contributing to the pathogenesis of RA remain unknown, both experimental and clinical findings indicate that an imbalance between pro- and anti-inflammatory cytokine activities favors the induction of autoimmunity, chronic inflammation, and the subsequent breakdown of the cartilage extracellular matrix (ECM) [1,2,3]. A schematic representation of the pathophysiological mechanisms of cartilage breakdown in RA has been presented in Figure 1.

Among the pro-inflammatory factors involved in the catabolic processes in RA, tumor necrosis factor-α (TNF-α) and interleukin 1β (IL-1β) play an active role in affecting both the quantity and quality of the cartilage ECM [2,3,4]. The ECM consists of two major components, one of which is a type II collagen (CII) fibrous network associated with small proteoglycans (PGs) and the other consists of proteoglycan aggregates composed of aggrecan, hyaluronan, and link protein [5,6,7]. The proteolytic cleavage of the major components of the cartilage matrix is mediated by proteases, including matrix metalloproteinases (MMPs) [2,3,5,6,7,8,9] and various types of disintegrin and metalloproteinase with thrombospondin motifs (ADAMTS) [2,3,6,7,8]. These proteases are synthesized by chondrocytes, synovial fibroblasts, macrophages, and neutrophils in response to inflammatory stimuli such as TNF-α and IL-1β [2,3]. Consequently, the breakdown of the collagenous and non-collagenous components of articular cartilage leads to the generation of cartilage-derived molecules and/or their fragments, which are released into the synovial fluid, where they may be filtered into the circulation and urine [7,10,11]. For this reason, soluble cartilage-derived biomarkers have been suggested to be powerful tools for the diagnosis, assessment of disease activity, prognosis, and personalized management of both osteoarthritis (OA) and RA [5,7,11,12,13]. Furthermore, due to the lack of non-invasive and effective methods for the early detection and evaluation of treatment outcomes in modern medicine [13], assessing biochemical markers in biological fluids can serves as a valuable supplementary indicator for imaging cartilage lesions in cases of RA. Currently, conventional radiography (CR) remains a simple, inexpensive, and widely used imaging technique for evaluating joint damage in patients with RA. However, early cartilage abnormalities are difficult to detect using this method [14]. On the other hand, modern imaging tools such as magnetic resonance imaging (MRI) and ultrasonography (US) exhibit higher sensitivity than CR in detecting local inflammation and structural damage, especially in the early stages of RA. Compared with MRI, US is more readily available, easier to perform, less expensive, and offers higher resolution. However, US has limitations with respect to visualizing an entire joint due to acoustic shadowing [13,14,15].

The ultimate goals of RA treatment are to relieve pain, control disease activity, and stop or slow the rate of joint damage, thereby preventing loss of function and disability [1]. The most well-known treatments for RA include the use of non-steroidal anti-inflammatory drugs (NSAIDs) and glucocorticoids, which are predominantly used for reducing pain and inflammation; conventional synthetic disease-modifying antirheumatic drugs (csDMARDs), which are administered as a first-line strategy for early RA; and biological therapies, which are designed to target specific components of the immune system [1]. In addition to these treatments, dietary polyphenols including phenolic acids, flavonoids, tannins, stilbenes, and lignans known for their antioxidant and anti-inflammatory characteristics [16], in addition to probiotics, specifically *Lactobacillus casei* or *Lactobacillus acidophilus*, are currently widely used as alternative and complementary therapies for the standard management of RA [17].

The administration of biological agents such as tumor necrosis factor α inhibitors (TNFαI) remain the most efficacious therapy for RA. Currently, there are five approved TNFαI: infliximab, (IFX), etanercept (ETA), adalimumab, (ADA), golimumab, (GLM), and certolizumab pegol (CZP) [1]. Recent data and meta-analysis assessing ADA, IFX, and ETA biosimilars in relation to RA treatment have confirmed that the biosimilar forms of these drugs are equivalent to their reference products [18,19]. Moreover, all available TNFαI similarly reduce disease activity and radiographic progression in RA patients and in animal models of arthritis [2,9,18,19,20,21]. However, the mechanism by which TNFαI prevent joint destruction and bone erosion is still unknown. Therefore, the main objective of this study was to evaluate the effect of long-term TNF-blocking therapy on circulating levels of biochemical markers of cartilage turnover to assess their potential diagnostic and prognostic applications for evaluating treatment effects, particularly in terms of monitoring structural joint damage among RA patients. Cartilage degradation was assessed by measuring serum levels of the collagen type II C-terminal cleavage neoepitope ((C2C) previously termed Col2-3/4C_long mono_) and the cartilage oligomeric matrix protein (COMP). The C-terminal propeptide of type II collagen (PIICP) was used as a marker of type II collagen synthesis. Additionally, serum matrix metalloproteinase-3 (MMP-3; stromelysin-1) was assessed as a laboratory disease activity index and a marker of synovial inflammation and cartilage breakdown. The associations between cartilage remodeling markers and clinical and laboratory variables of disease activity with respect to patients with RA were also examined in this study.

## 2. Materials and Methods

### 2.1. Patients and Samples

In this study, a total of 50 female patients with RA were included, who were classified according to the 1987 or 2010 American College of Rheumatology/European League Against Rheumatism (EULAR) criteria [22,23]. All women failed to achieve remission despite using at least 2 csDMARDs, and the baseline 28-joint disease activity score based on the erythrocyte sedimentation rate (DAS28-ESR) at baseline was >5.1. Patients were not eligible to participate if they met any of the following exclusion criteria: (1) age <18 years; (2) previous use of biological agents; (3) pregnant, breastfeeding, or planning to conceive; (4) presence of inflammatory rheumatic or autoimmune joint disease other than RA; (5) afflicted by acute or recent infectious disease; (6) history of cardiac, renal, psychiatric, endocrine, metabolic, or hepatic disease, including with malignancy; or (7) suffered from chronic alcoholism. None of the patients received chondroprotective treatments, such as glucosamine sulfate, chondroitin sulfate, hyaluronic acid, or collagen hydrolysate, that potentially interfere with cartilage metabolism. In addition, none of the enrolled subjects received bisphosphonates or hormone replacement therapy. First-line therapy with anti-TNF-α inhibitors was administered in combination with methotrexate (MTX; 25 mg/week) over a 15-month period. The biological agents were administered subcutaneously at the recommended doses for RA, namely, 40 mg every 2 weeks for ADA; 50 mg every week for ETA; 400 mg at weeks 0, 2, and 4 and then 200 mg every 2 weeks for CZP; and 50 mg once a month for GLM. Patients were also prescribed prednisone (≤7.5 mg/day) and folic acid (5 mg/day). Administration of concomitant medications remained unchanged throughout the study duration. 

Patients were evaluated with respect to their therapeutic responses at baseline and 3, 9, and 15 months after starting anti-TNF-α treatment. Table 1 presents the demographic and clinical data variables of the analyzed female RA patients during the 15-month TNFαI therapy, which were obtained from our previous investigations [24,25].

A control group consisting of 26 age-matched healthy female volunteers from the Medical University of Silesia in Katowice, Poland, was established. The subjects were selected based on their medical histories and laboratory tests. Women enrolled in this study had not been afflicted with any diseases requiring hospitalization or recent surgical procedures within the past 3 years. Moreover, all volunteers underwent normal morphological and biochemical analysis. None of the participants had been taking glucocorticoids or any other medications known to affect cartilage metabolism, and they had no history of alcohol abuse. Women with a body mass index (BMI) < 25 kg/m^2^ were deliberately chosen.

Venous blood samples were obtained from all participants in the morning (7:00 a.m.–9:00 a.m.) after overnight fasting. Routine laboratory parameters, including hematology and biochemical profiles, were analyzed immediately. Additional aliquots of blood serum samples were frozen and stored at −80 °C until further analysis. Clinical and laboratory tests were conducted prior to the initiation of TNFαI therapy and at months 3, 9, and 15 during the follow-up period. The levels of cartilage remodeling markers (C2C, PIICP, and COMP) and MMP-3 were determined at baseline and after 15 months of TNFαI treatment.

Following approval from the Ethical Committee of the Medical University of Silesia in Katowice (KNW/0022/KB/56/I/12/13), patients identified for participation were briefed on the purpose of the study. Signed consent forms were obtained from all participants, and the research was carried out in accordance with the principles outlined in the Declaration of Helsinki. 

### 2.2. Clinical Assessment of Anti-TNF-α Treatment Response

DAS28-ESR, ESR, C-reactive protein (CRP), and MMP-3 were used as clinical and laboratory markers to monitor the disease activity among RA patients during the 15-month anti-TNF-α treatment period. DAS28-ESR, which has a score ranging from 0 to 9.4, was calculated according to a formula that incorporates the number of tender and/or swollen joints out of 28 (TJC28/SJC28), ESR (mm/h), and patients’ global assessment of their health on a 100 mm visual analogue scale (VAS). The methods used for the measurement of biologic markers of inflammation, i.e., ESR and CRP, were previously described in our earlier investigation [24,25]. Among the laboratory indices for RA disease activity, MMP-3 was evaluated at baseline and after 15 months of biological therapy. Serum levels of MMP-3 were analyzed using a quantitative sandwich enzyme-linked immunosorbent assay (ELISA) developed by Ray Biotech Life, Inc. (Peachtree Corners, GA, USA), according to the manufacturer’s instructions. The analytical sensitivity was estimated to be 300 pg/mL. All samples were tested in one day; thus, inter-assay variation was insignificant. The intra-assay coefficient of variation (CV) was <10%.

Patients who did not respond well to TNFαI treatment were excluded from the study. An adequate treatment response was defined—according to the Polish National Health Fund Therapeutic Programs (B.33 or B.45)—as a reduction in DAS28-ESR > 1.2 after the first 3 months of TNF-blocking therapy and a further reduction in DAS28-ESR by 1.2 noted in subsequent medical examinations performed 9 and 15 months after the administration of the first dose of TNFαI.

### 2.3. Immunoassay of Biochemical Cartilage Markers

To evaluate the effect of TNF-α inhibition on cartilage metabolism, the levels of biochemical markers of cartilage turnover were evaluated in serum samples collected at baseline and 15 months after initiating TNFαI therapy. Cartilage degradation was analyzed based on serum levels of C2C and COMP using commercially available ELISA kits purchased from BioVendor R&D (Brno, Czech Republic) and IBEX Pharmaceuticals, Inc. (Montreal, QC, Canada), respectively. Serum levels of cartilage synthesis marker, PIICP, were determined using a sandwich ELISA developed by Cloud-Clone Corp. (Katy, TX, USA). Testing of all samples in duplicate was completed in one day to eliminate the influence of inter-assay variation. The CVs for C2C and PIICP were <10%, they were and 4–8% for COMP. 

Additionally, to assess the relative balance between type II collagen matrix synthesis and degradation, the C2C/CPII ratios were calculated.

### 2.4. Statistical Analysis

The obtained results were subjected to statistical analysis using TIBCO Software, Inc. (Palo Alto, CA, USA), version 13.3; StatSoft Poland Sp. z o. o. 2022. The normality of the data distribution was evaluated using the Shapiro–Wilk test. Continuous variables were presented as means ± SD if normally distributed or as the median and interquartile ranges (25th–75th percentile) otherwise. The homogeneity of variance was assessed using Levene’s test. The significance of the differences between the parameters obtained from the RA patients and healthy subjects was determined using independent-samples Student’s t test or the Mann–Whitney U test. The paired Student’s t test or Wilcoxon’s rank-sum test was used to compare changes in parameters within each RA patient before and after 15 months of anti-TNF-α treatment. A ***p***-value less than 0.05 was considered significant. Moreover, evaluation of clinical data (SJC28, TJC28, VAS, and DAS28-ESR) and inflammatory parameters (ESR and CRP) was carried out using repeated measures analysis of variance (RM-ANOVA) Friedman’s test. Post-hoc analyses, which were performed in cases of significant differences between subgroups, relied on the Mann–Whitney U test with a *p*-value obtained after applying Bonferroni correction (*p* < 0.05/six possible comparisons). Spearman’s rank correlation coefficient was used to evaluate the relationship between selected biomarkers of cartilage turnover (C2C, PIINP, C2C/PIINP ratios, and COMP) and age, duration of disease, and indicators of disease activity among women with RA. A linear multiple regression analysis of the variables was performed. Receiver Operating Characteristic (ROC) analysis was used to evaluate and compare the performance of each cartilage turnover biomarker. A *p*-value less than 0.05 was considered significant.

## 3. Results

### 3.1. Effects of TNFαI Therapy on Systemic Inflammation and Disease Activity

**Figure 2:**
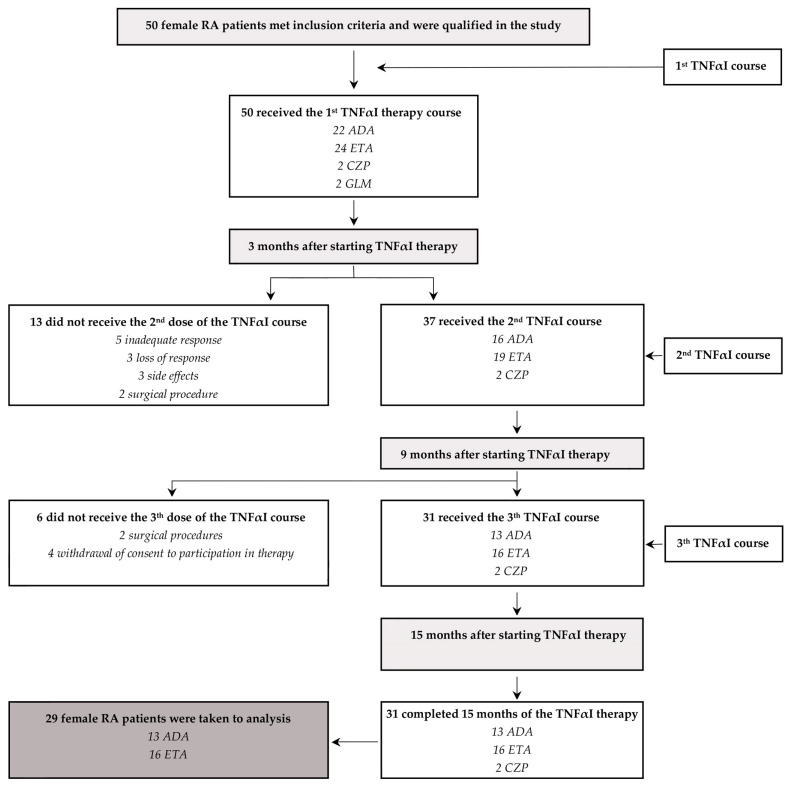
A flowchart describing the female patients with RA receiving treatment with TNFαI and the primary reasons why patients dropped out of this study. ADA, adalimumab; ETA, etanercept; CZP, certolizumab pegol; GLM, golimumab; RA, rheumatoid arthritis; TNFαI, tumor necrosis factor-α inhibitors.

Among the 50 female RA patients receiving their first TNF-α inhibitors, 31 patients (62%) continued with the first inhibitor for 15 months, while 19 (38%) completely discontinued their use and were excluded from our analysis (Figure 2). The administration of TNFαI was discontinued due to the following reasons: inadequate response (five patients), loss of response (three patients), side effects (three patients), surgical procedures (four patients), and withdrawal of consent for participation in the therapeutic trial (four patients). Moreover, due to the very small size of the cohort, two patients who completed 15 months of treatment with CZP were excluded from the final analysis. Therefore, a total of 29 female RA patients who continued the anti-TNF-α treatment with ETA or ADA for 15 months were included in the study (Table 2, Figure 2).

During TNF-α inhibition, a significant clinical improvement was observed for all the RA patients. As expected, clinical parameters, such as TJC28, SJC28, VAS, and DAS28-ESR, showed significant reductions after 3, 9, and 15 months of TNFαI therapy compared to the baseline values (Table 1). Furthermore, besides ESR and CRP, the levels of other markers of systemic inflammation, i.e., MMP-3, decreased significantly after 15 months of anti-TNF-α treatment (*p* < 0.001; Table 1). It was observed that 83% of the patients achieved remission (defined as a DAS28 value ≤ 2.6), while 17.24% presented at least low disease activity (defined as a DAS28 value < 3.2) upon the 15th month of treatment. According to the EULAR response criteria [26], all 29 female RA patients (100%) presented a good response (DAS28-ESR improvement > 1.2) after 3 months, and this effect persisted up to month 15.

Table 1 presents the demographic, clinical, and biochemical data (excluding serum levels of MMP-3) of the RA patients during the 15-month TNFαI therapy, which were obtained in our previous investigations [24,25].

### 3.2. Effects of TNFαI Therapy on Cartilage Remodeling Markers

**Figure 3:**
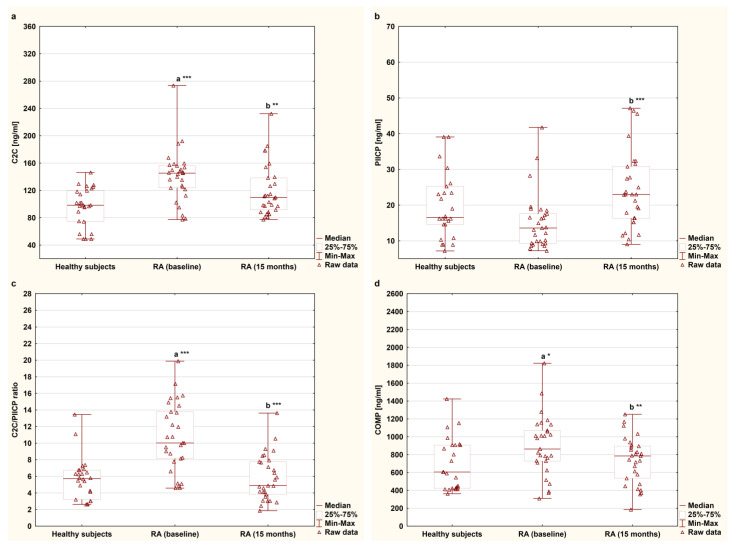
Circulating levels of cartilage remodeling markers: C2C (**a**), PIICP (**b**), C2C/PIICP ratios (**c**), and COMP (**d**) levels in women with rheumatoid arthritis at baseline and after 15 months of anti-TNF-α therapy and in healthy subjects. ^a^ statistically significant differences compared to healthy subjects; ^b^ statistically significant differences compared to baseline values; * *p* < 0.05; ** *p* < 0.01; *** *p* < 0.001. C2C, collagen type II C-terminal cleavage neoepitope; COMP, cartilage oligomeric matrix protein; PIICP, C-terminal propeptide of type II collagen; RA, rheumatoid arthritis; TNF-α, tumor necrosis factor-α.

The serum concentrations of C2C, PIICP, and COMP and the C2C/PIICP ratios of the female RA patients undergoing TNFαI therapy and of the healthy subjects are presented in Figure 3a–d. Among the analyzed cartilage degradation markers, serum levels of C2C and COMP were significantly higher in women with RA before anti-TNF-α therapy [145.26 (123.82–156.00) ng/mL, 863.45 (728.05–1071.20) ng/mL, respectively] compared to those of the controls [98.42 (74.90–119.71) ng/mL, 605.67 (423.66–906.72) ng/mL, respectively], (*p* < 0.001 and *p* < 0.05, respectively; Figure 3a,d). In addition, a concomitant reduction in C2C and COMP levels was observed after TNFαI therapy compared to the baseline values (141.27 ± 38.54 ng/mL to 119.43 ± 37.77 ng/mL and 898.15 ± 336.16 ng/mL to 733.89 ± 263.24 ng/mL, respectively, both of which corresponded to *p* < 0.01; Figure 3a,d). Furthermore, at 15 months, the patients had values of both C2C and COMP levels that were comparable to those of the healthy subjects [109.90 (91.54–138.33) ng/mL vs. 98.42 (74.90–119.71) ng/mL, 785.30 (535.15–895.48) ng/mL vs. 605.67 (423.66–906.72) ng/mL, respectively) (*p* = 0.084 and *p* = 0.487, respectively; Figure 3a,d). Regarding the marker of type II collagen synthesis, there were no significant differences in PIICP levels between the women with RA before TNFαI therapy and the control group [13.59 (9.39–17.58) ng/mL vs. 16.54 (14.59–26.26) ng/mL], (*p* = 0.055; Figure 3b). Interestingly, serum levels of PIICP increased after 15 months of treatment with TNFαI (15.21 ± 7.86 ng/mL to 24.04 ± 10.74 ng/mL) (*p* < 0.001; Figure 3b), but they were still not different from the control values [16.54 (14.59–26.26) ng/mL], (*p* = 0.146; Figure 3b). Based on these findings, it was determined that the C2C/PIICP ratios significantly decreased from the baseline levels of 10.74 ± 4.12 to 5.85 ± 2.78 after 15 months during the TNFαI treatment (*p* < 0.001; Figure 3c), reaching the same value as that of the healthy population [5.72 (3.20–6.75)], (*p* = 0.719; Figure 3c). 

**Figure 4:**
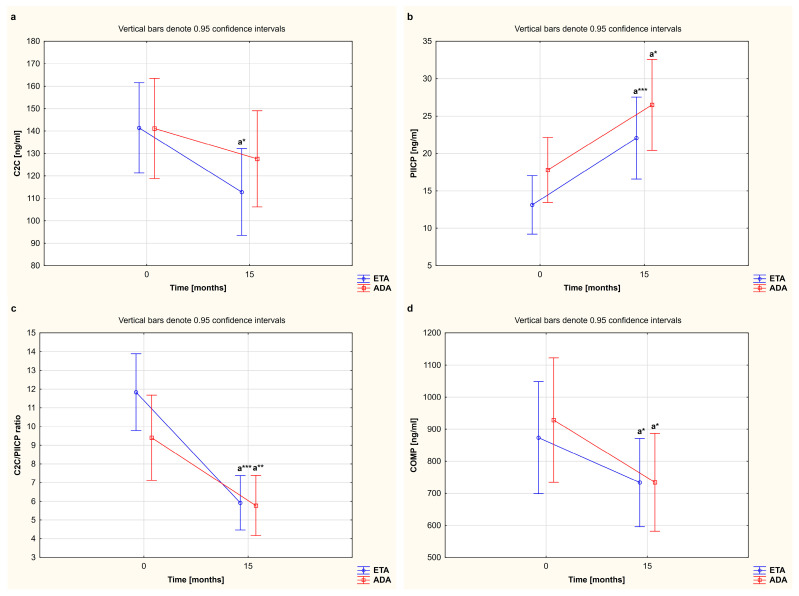
The effect of the type of TNF-α inhibitor used on circulating levels of cartilage remodeling markers, i.e., C2C (**a**), PIICP (**b**), C2C/PIICP ratios (**c**), and COMP (**d**), in women with rheumatoid arthritis during 15-month therapy. ^a^ Statistically significant differences compared to baseline. * *p* < 0.05; ** *p* < 0.01 or *** *p* < 0.001 compared to the baseline. ADA, adalimumab; C2C, collagen type II C-terminal cleavage neoepitope; COMP, cartilage oligomeric matrix protein; ETA, etanercept; PIICP, C-terminal propeptide of type II collagen; RA, rheumatoid arthritis; TNF-α, tumor necrosis factor-α.

In our study, we also compared alterations in serum levels of cartilage remodeling markers in female RA patients who completed a 15-month TNFαI therapy with ETA or ADA (Table 2, Figure 2). The results regarding the observed serum levels of C2C, PIICP, and COMP, depending on the type of TNFαI used, are reported in Figure 4a,b,d. Overall, for women with RA who received ETA or ADA, both TNF-α-blocking agents led to a significant decrease in serum levels of COMP (873.52 ± 334.26 ng/mL to 733.56 ± 317.77 ng/mL and 928.46 ± 349.58 ng/mL to 734.30 ± 188.33 ng/mL, respectively) (both *p* < 0.05; Figure 4d). We also found that patients responding to etanercept presented a significant decrease in serum levels of C2C and an increase in serum levels of PIICP (141.39 ± 43.72 ng/mL to 112.79 ± 29.99 ng/mL and 13.12 ± 6.37 ng/mL to 22.05 ± 9.82 ng/mL, respectively) (*p* < 0.05 and *p* < 0.001, respectively; Figure 4a,b). On the other hand, serum levels of C2C were not significantly changed by the treatment with ADA (141.12 ± 32.80 ng/mL vs. 127.59 ± 45.52 ng/mL); however, serum levels of PIICP increased after 15 months of therapy (17.79 ± 8.97 ng/mL to 26.50 ± 11.69 ng/mL) (*p* = 0.266 and *p* < 0.05, respectively; Figure 4a,b). Interestingly, the C2C/PIICP ratios also decreased in both the etanercept- and adalimumab-treated patients (11.84 ± 3.56 to 5.92 ± 2.71, 9.40 ± 4.50 to 5.77 ± 2.97, respectively) (*p* < 0.001 and *p* < 0.01, respectively; Figure 4c). Meanwhile, there were no significant changes in the serum levels of the analyzed cartilage biomarkers, i.e., C2C, PIICP, COMP, and C2C/PIICP ratios, depending on the type of TNF-α inhibitor used (*p* = 0.335, *p* = 0.952, *p* = 0.561, and *p* = 0.134, respectively; Figure 4a–d).

### 3.3. Correlations between Cartilage Remodeling Markers and Demographic Parameters as Well as Clinical and Laboratory Indicators of Disease Activity among Female RA Patients Receiving TNFαI Therapy 

**Figure 5:**
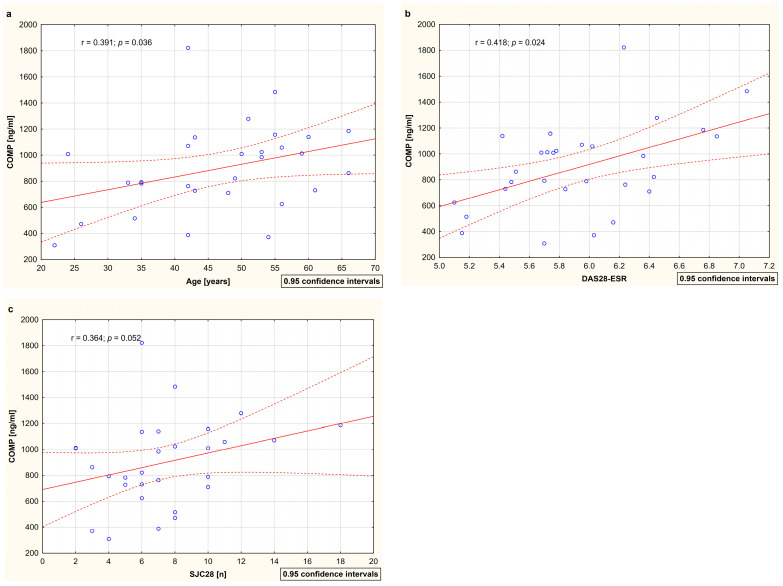
The relationships between circulation levels of COMP and age (**a**), disease activity score (**b**), and swollen joint count (**c**) among female RA patients at baseline. Data are expressed as r values (correlation coefficient) determined according to Spearman rank correlation. Correlations were considered significant at *p* < 0.05. COMP, cartilage oligomeric matrix protein; DAS28-ESR, 28-joint disease activity score based on erythrocyte sedimentation rate; ESR, erythrocyte sedimentation rate; RA, rheumatoid arthritis; SJC28, swollen joint count out of 28 joints.

The analysis of the relationships between cartilage remodeling markers (C2C, PIICP, C2C/PIICP, and COMP) and demographic parameters (age and disease duration) as well as clinical (DAS28-ESR, SJC28, TJC28, and VAS) and laboratory indicators (ESR, CRP, and MMP-3) of disease activity with respect to RA patients both before and after 15 months of TNFαI therapy is presented in Table 3. No significant correlations were found between type II collagen turnover markers (i.e., C2C, PIICP, and C2C/PIICP ratios) and any of the evaluated parameters. A positive correlation between serum COMP levels and the age of the RA patients was found (r = 0.391; *p* < 0.05) (Table 3, Figure 5a). Moreover, before the first dose of TNFαI, serum COMP levels significantly correlated with DAS28-ESR (r = 0.418; *p* < 0.05) and tended to correlate with SJC28 (r = 0.364; *p* = 0.05) (Table 3, Figure 5b,c). After 15 months of TNFαI therapy, these correlations were not significant (Table 3). The COMP values did not correlate significantly with the levels of all biomarkers of inflammation, i.e., ESR, CRP, and MMP-3, both at baseline and after treatment (Table 3). No correlations between serum COMP and the disease duration and the TJC28 and VAS of the patients were noted at any time point (Table 3).

Furthermore, we observed no correlations between the patients’ ages, disease duration, all markers of RA activity, and the serum levels of MMP-3 both before and after TNFαI therapy.

Additionally, a multiple linear regression analysis was performed to examine the serum COMP levels in patients with RA at baseline (Table 4). This analysis showed that DAS 28-ESR was the only variable in the model that had a significant impact on the levels of COMP in female RA patients at baseline (β = 287.74, *p* = 0.022, and R^2^ model = 0.25).

### 3.4. Determination of the Diagnostic Utility of Circulating Markers of Cartilage Turnover for the Evaluation of Anti-TNF-α Therapeutic Response

**Figure 6:**
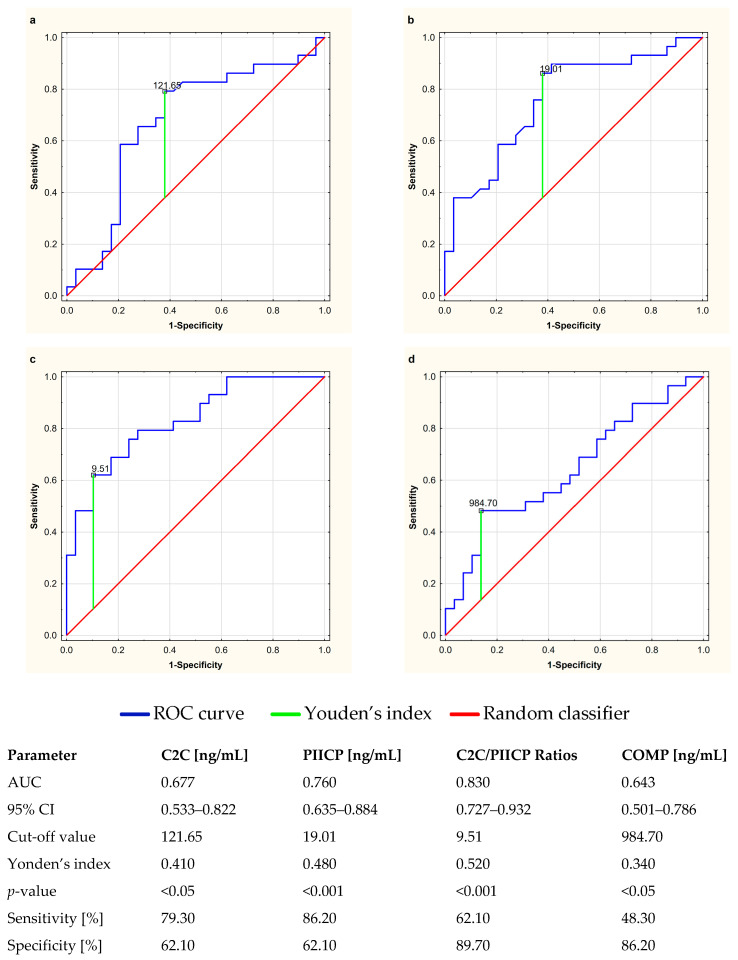
Receiver operating characteristic (ROC) curves used to determine the clinical usefulness of cartilage turnover biomarkers, i.e., C2C (**a**), PIICP (**b**), C2C/PIICP ratios (**c**) and COMP (**d**), and their diagnostic power in the evaluation of anti-TNF-α therapeutic response in relation to rheumatoid arthritis. The diagonal segment is the reference line. AUC, area under the ROC curve; C2C, collagen type-II C-terminal cleavage neoepitope; CI, confidence interval; COMP, cartilage oligomeric matrix protein; PIICP, procollagen type II C-terminal propeptide; Youden’s index for the ROC curve.

The clinical usefulness of serum cartilage biomarkers and their diagnostic power in the evaluation of anti-TNF-α therapeutic response among RA patients was determined by assessing the area under the receiver operating characteristic curve (AUC). The ROC curves for C2C, PIICP, COMP, and C2C/PIICP ratios are presented in Figure 6a–d. The C2C levels demonstrated a sensitivity of 79.30% and a specificity of 62.10%, with a cut-off value of 121.65 ng/mL (Figure 6a). The PIICP values presented a sensitivity of 86.20% and a specificity of 62.10%, with a cut-off value of 19.01 ng/mL (Figure 6b). Furthermore, the C2C/PIICP ratios demonstrated a sensitivity of 62.10% and a specificity of 89.10%, with a cut-off value of 9.51 (Figure 6c), while the serum levels of COMP presented a sensitivity of 48.30% and a specificity of 86.20%, with a cut-off value of 984.70 ng/mL (Figure 6d). Moreover, the largest area under the ROC curve was obtained for the C2C/PIICP ratios (AUC: 0.830, 95% CI: 0.727–0.932, *p* < 0.001) (Figure 6c). The *p*-values of the AUC for all the biomarkers of cartilage turnover were less than 0.05 and statistically significant.

## 4. Discussion

### 4.1. Type II Collagen Biomarkers (C2C, PIICP, and C2C/PIICP Ratios) in Female RA Patients Undergoing TNFαI Therapy and in Healthy Subjects

The progressive destruction of articular cartilage, resulting from an imbalance between anabolic and catabolic processes, is an underlying problem in the pathogenesis of rheumatoid arthritis and osteoarthritis [3,9,27]. Due to articular cartilage damage, key structural components of the extracellular matrix are lost and released into body fluids as ‘biomarkers’, which can be detected by sensitive immunoassays [7,12,13]. Alterations in collagen type II metabolism occur prior to detectable radiographic changes, making this peptide a specific and early marker of arthritic joint diseases [5,12]. The excessive and progressive cleavage of CII by members of the collagenase subfamily of MMPs such as MMP-1, -8, -13, and -14 has been observed in RA, psoriatic arthritis (PsA), and the early stages of OA [3,5,8,27,28]. Consistent with our results, higher levels of C2C have been observed in the sera from RA patients compared to those of the corresponding controls [29]. Moreover, it has been documented [30,31] that elevated baseline C2C levels in RA patients are associated with the progression of radiographic joint damage, indicating that circulating C2C may be useful for predicting disease progression and monitoring clinical outcomes after the aggressive anti-inflammatory treatment of RA. It is well known that the loss of structural cartilage components during active arthritis usually leads to increased cartilage turnover, involving increased matrix synthesis. During the synthesis of new molecules of type II collagen, C-terminal propeptides are cleaved by specific proteases and released into biological fluids, and their levels are believed to reflect CII synthesis. The rate of PIICP release is proportional to the rate of current CII synthesis, as the propeptide has a half-life of approximately 18 h [5]. Since C-terminal propeptides are predominantly found in cartilage and intervertebral discs, it is likely that most synovial and serum PIICP originate from articular cartilage [32]. In the present study, no significant differences were found in the serum PIICP levels between the female RA patients before treatment with TNFαI and those of the healthy subjects. These results are in line with previous studies conducted by Kopeć-Mędrek et al. [33], who reported that there were no differences between the serum levels of PIICP in women with RA before IFX treatment and those of a control group. Conversely, other studies have demonstrated significantly higher levels of PIICP in cartilage tissue extract and synovial fluid from patients with early RA compared to healthy controls [34], although a decrease was observed in another study [28]. Moreover, these increases in CPII content were accompanied by an increase in serum PIICP in RA patients [34,35], without the presentation of significant differences between aggressive and non-aggressive disease [30,35]. The reasons for these discrepancies cannot be easily explained but may result from differences in the populations’ characteristics. Our female patients were characterized by long-standing disease (with a median disease duration of 5 years (interquartile range of 3–11)) and active RA with a DAS28 > 5.1. The lack of significant changes in serum PIICP levels between the female RA patients and the control group may be attributed to the more severe cartilage damage in the late stage of RA compared to that of early RA, thus causing the synthesis ability to decrease markedly. This suggestion is supported by the observed increased C2C/PIICP ratio, which serves as an indicator of type II collagen turnover in RA-afflicted women that differentiates them from healthy individuals at the same age. 

In conclusion, maintaining a homeostatic balance between the synthesis and degradation of key matrix molecules, such as type II collagen, represents a critical factor of articular cartilage integrity. Thus, the extensive breakdown of CII fibrils combined with inadequate synthesis within the background of RA leads to the loss of the physical properties of cartilage. This loss is accompanied by swelling, warmth, joint pain, and decreased mobility. Therefore, early intervention with anti-TNF-α agents aiming to limit the progression of cartilage destruction is critical for mitigating patients’ physical disability and improving their quality of life. As anticipated, the 15-month anti-TNF-α treatment conducted in this study proved to be effective with respect the RA patients, significantly reducing DAS28-ESR and the levels of all inflammation biomarkers, namely, ESR, CRP, and MMP-3. In line with our findings, previous studies have reported a decrease in inflammation biomarkers during TNFαI treatment, including routine clinical parameters such as ESR and CRP [33,36,37] as well as MMP-3 [36,37,38]. The anti-inflammatory effects of TNFαI were associated with an improvement in articular cartilage turnover, as assessed according to the serum levels of type II collagen synthesis and degradation biomarkers (PIICP and C2C, respectively). Indeed, higher serum levels of PIICP and lower levels of C2C neoepitope were observed when the patients were treated with TNFαI. Moreover, these changes were accompanied by significantly reduced C2C/PIICP ratios, suggesting an improvement in cartilage remodeling balance, mainly due to the inhibition of cartilage loss. Additionally, when comparing all the parameters studied, no differences were found between the use of etanercept and adalimumab, suggesting that they have similar protective effects on cartilage metabolism in RA patients. There are only a few data in the literature regarding the effect of anti-TNF-α treatment on serum PIICP and C2C levels in RA patients. Both Mullan et al. [31] and Kopeć-Mędrek et al. [33] indicated in their studies that the administration of TNFαI blockade to RA patients had no effect on circulating levels of PIICP. Additionally, in line with our results, Mullan et al. [31] confirmed that serum C2C levels were significantly downregulated following 1-year therapy with TNFαI (IFX, ADA, or ETA). Moreover, these researchers [31] demonstrated that the decrease in C2C levels at 1 month of TNFαI therapy were significantly predictive of clinical remission. Interestingly, this effect was independent of the change in disease activity and inflammation indices of the RA patients [31]. Similarly, in our study, no significant correlations between C2C levels and clinical (DAS-28-ESR) and laboratory (ESR and CRP) indicators of disease activity in RA patients were observed. These findings indicate that measurements of serum C2C levels taken before initiating anti-TNF-α therapy and after its initiation may provide additional information regarding the radiographic prognosis of patients with RA that is not provided by successive assessments of disease activity. The outcomes of this study also correspond with the results reported by Niki et al. [36]. They demonstrated that combination treatment with infliximab and MTX significantly reduced C2C/PIICP ratios during the 54-week observation period in patients with early RA, and this decrease was correlated with changes in CRP, DAS28, radiographic progression, and patient function (HAQ). However, they did not find any effect of therapy on the C2C/PIICP ratios in the established RA patients [36]. Finally, Briot et al. [39] showed that effective 2-year treatment of spondyloarthropathy patients with etanercept was associated with decreased serum levels of C2C and increased serum levels of PIICP. Taken together, it seems that the reduction in C2C/PIICP ratios after successful treatment with TNF-α blockers may reflect a protective effect of TNFαI on cartilage metabolism, which primarily occurs through the suppression of cartilage-degrading enzymes, such as MMPs and ADAMTSs. Indeed, TNFαI therapy has been found to downregulate the levels of various MMPs in the serum of RA patients [40,41,42,43,44,45]. Additionally, in our previous studies [25,46], we documented the beneficial effects of TNF-α blockade on serum levels of MMP-9, as well as ADAMTS-4 and ADAMTS-5, in female RA patients. In the present study, we observed a statistically significant reduction in the serum concentration of MMP-3 in response to anti-TNF-α treatment. This is likely associated with the inhibitory effect of anti-TNF-α agents on articular chondrocytes and synovial tissue fibroblasts, which are constant sources of MMPs and ADAMTS. As previously reported by Zwerina et al. [47], TNF-α blockade with infliximab gradually decreases the expression of MMP-3, -9, and -13 in articular chondrocytes of human TNF–transgenic mice with polyarthritis (hTNFtg). Moreover, IFX treatment also leads to a marked reduction in proteoglycan loss from the cartilage matrix, as quantitatively assessed via the toluidine blue staining of articular cartilage [47]. Similarly, the immunohistochemical staining of synovial tissues from RA patients showed reduced expression of MMP-3 and a decreased number of MMP-3-positive staining cells after adalimumab therapy [48]. Taken together, the present findings suggest that TNFαI counteract the progressive cartilage degeneration associated with RA by inhibiting MMP-3 expression.

### 4.2. MMP-3 as a Marker of Disease Activity and Cartilage Breakdown in Female RA Patients Undergoing TNFαI Therapy and in Healthy Subjects

It is well known that MMP-3 represents a potential indicator of early diagnosis and the activity of RA [2,9]. During cartilage remodeling in RA, MMP-3 plays a major role in the breakdown of various cartilage matrix components, including the aggrecan core protein, laminin, fibronectin, and collagen types IV, VII, IX, and XI. Although MMP-3 is not directly collagenolytic, this enzyme activates other pro-MMPs, such as pro-MMP-1, pro-MMP-7, pro-MMP-8, pro-MMP-9, and pro-MMP-13, and is the most important cause of collagen type II damage [3,6,8,9,49]. Several studies have indicated that serum MMP-3 levels significantly correlate with the levels produced by the synovium and thus reflect synovial inflammation and cartilage turnover in inflammatory joint diseases. The baseline serum levels of MMP-3 are significantly higher in RA patients with high-progression, making MMP-3 an early predictor of progressive joint erosion [28,45,50,51,52,53,54]. These findings have encouraged some researchers to recommend MMP-3 monitoring in the routine assessment of prognosis and the prediction of treatment response in cases of RA [55]. Our study has shown that circulating levels of MMP-3 were much higher in women with RA before TNFαI treatment than in healthy subjects of the same age. Moreover, as mentioned earlier, after 15 months of treatment, the serum levels of MMP-3 decreased but were still higher than those in the controls. Similar results were found in previous studies [36,37,38,40,41,42,43,44,52,54,56]. However, only Sun et al. [42] reported significantly lower MMP-3 levels in RA patients treated with TNFαI, suggesting that serum MMP-3 may be a useful biomarker for monitoring anti-TNF-α treatment efficiency, whereas these levels did not correlate with the disease activity indices of RA. Furthermore, contrary to our results, many researchers found a linear correlation between baseline serum MMP-3 levels and DAS28, CRP and/or ESR, which showed that MMP-3 is a useful clinical marker of inflammation and thus an indicator of RA activity [37,38,43,44,52,54,57]. The lack of correlation between MMP-3 and markers of RA activity in our study may be due to the relatively small number of patients or the participants’ sex. Our subject population comprised only women with RA. Notably, serum MMP-3 levels are affected by sex difference. Ribbens et al. [57] demonstrated that serum levels of MMP-3 are significantly higher in males than in females. Furthermore, Natoli et al. [58] showed that higher gene and protein expression of MMP-3 was induced by male sex testosterone.

### 4.3. COMP as a Non-Collagenous Biomarker of Cartilage Breakdown in Female RA Patients Undergoing TNFαI Therapy and in Healthy Subjects

In the present study, we also analyzed the effect of TNFαI therapy on serum levels of COMP in female RA patients. COMP is a pentameric member of the thrombospondin family and a non-collagenous ECM glycoprotein mainly present in the articular cartilage and, in smaller amounts, in tendons, menisci, and the synovial membrane. In adult cartilage with slow collagen turnover, COMP is located primarily in the interterritorial matrix, where it interacts with the COL3 and NC-4 domains of type IX collagen, stabilizing the collagen fiber network [9,11,59,60,61]. During cartilage erosion, COMP fragments are released first into synovial fluid and then into the blood; thus, it is believed that serum COMP reflects the degree of cartilage breakdown [7,12]. Elevated levels of serum and synovial COMP have been detected in various forms of arthritis, such as RA, OA [35,62,63], and PsA [64]. Moreover, Anderson et al. [65] revealed that a high serum level of COMP in patients with early RA at the first 3 months after diagnosis predicted significant joint damage progression over the next 5 years. These results are in line with the studies conducted by Saghafi et al. [63] and Sakthiswary et al. [66]. Saghafi et al. [63] reported that there was a significant positive correlation between serum COMP levels and disease severity in both early RA and late RA, whereas Sakthiswary et al. [66] showed that there was an inverse relationship between bilateral sonographic knee cartilage thickness and COMP levels among the RA patients and the controls. Furthermore, Liu et al. [67] found elevated levels of serum COMP in patients with active RA when compared to patients with RA in remission. Taken together, these results lend credence to the notion that serum COMP levels reflect damage of articular cartilage in RA patients. In line with this suggestion, we have noted higher serum COMP levels in women with active RA prior to starting anti-TNF-α therapy than in the control group, which may indicate an activated destructive process and significant joint damage in RA patients. Moreover, effective 15-month anti-inflammatory treatment with TNFαI led to a significant decrease in COMP levels compared to the values observed in the healthy subjects. Several studies have reported consistent findings [36,37,38,68]. Niki et al. [36] found a significant decrease in the serum levels of COMP after 54 weeks of infliximab combination therapy with MTX in patients with established RA. Kawashiri et al. [37] also observed this positive effect in a study incorporating RA patients who achieved remission after 6 months of etanercept combined therapy with csDMARDs. Our results are also in line with previous studies by Crnkic et al. [68] and den Broeder et al. [38]. Crnkic et al. [68] described a decrease in serum COMP levels in patients with RA after a 3-month treatment with either infliximab or etanercept. In turn, den Broeder et al. [38] examined the concentration of serum COMP in 47 patients with active RA at baseline and 2 years after starting adalimumab monotherapy. The authors demonstrated that the adalimumab-therapy-triggered decrease in serum COMP levels was more significant in the patients with radiological progression as determined through radiographs of the hands and foot joints than those with a stable radiological course, suggesting that COMP levels may also predict small joint damage in RA [38]. On the contrary, Hjeltnes et al. [69] and Morozzi et al. [70] showed that effective long-term anti-TNF-α therapy did not change serum COMP levels in RA patients. The possible explanation for these discrepancies could relate to differences in the ethnicity, gender, age, and exercise levels of the patients; disease activity and duration; administered dosages of csDMARDs and steroids; and the relatively low number of patients in both our study and some other studies. Finally, we found that serum COMP levels correlated significantly with disease activity based on DAS28-ESR and the age of patients with RA at baseline. These findings are consistent with the results obtained by other authors [38,62,66,71]. However, Hjeltnes et al. [69] and Morozzi et al. [70] reported that there was no significant correlation between serum COMP and RA activity at baseline or during treatment with TNFαI. On the other hand, in our study, the analyzed inflammatory markers, such as ESR and CRP, did not present any significant relationship with the level of serum COMP in female RA patients, which is in agreement with the results of previous studies. Hjeltnes et al. [69] did not find any significant associations between the inflammatory markers ESR and CRP and changes in serum COMP levels in patients with RA both before and during 6 months of treatment with MTX or MTX in combination with TNFαI. A similar independent relationship between ESR or CRP and COMP levels was previously described regarding patients with RA treated with adalimumab [38,70], etanercept, or infliximab [68], which may indicate that the cartilage destruction process is not directly linked to inflammation in RA. This hypothesis is supported by the results of Niki et al. [36], who demonstrated that there were higher serum levels of COMP in patients with established RA and lower pre-treatment CRP levels than those in patients with early RA and elevated pre-treatment CRP levels. However, this finding contrasts with the findings of a few studies that showed a significant positive correlation between serum COMP levels and inflammatory indices such as CRP and/or ESR [37,62,66]. In summary, our results further confirm the conclusion that serum COMP levels are highly specific non-inflammation-related markers reflecting disease activity and the cartilage degradation process in RA. 

This study has some potential limitations. First, the group of women with RA was relatively small, but it was carefully selected according to the Polish National Health Fund Therapeutic Programs that employ TNF-blockers (B.33 or B.45). Additionally, the effect of CZP on the levels of cartilage turnover markers was not assessed because only two patients were treated with this drug and completed 15 months of TNFαI treatment. This relatively small number limited the potential for detecting smaller changes in variables, and although 29 patients responded well to TNFαI treatment, this group was heterogeneous, consisting of pre- and postmenopausal women. Third, we did not examine the effect of ADA or ETA on the cartilage turnover marker levels in patients with seropositive vs. seronegative RA because only three of the women were RF negative and five were anti-CCP negative. Another limitation of this study is the lack of radiographs for comparison with the changes in serum cartilage turnover markers.

## 5. Conclusions

We report that the successful anti-inflammatory treatment of RA with TNFαI exerts a chondroprotective effect on cartilage turnover, improving the ratios of type II collagen resorption/formation (C2C/PIICP) in the evaluation of anti-TNF-α therapeutic response in RA patients. Additionally, baseline serum COMP was found to be a marker that reflects the current disease activity of RA. However, further studies must be conducted to definitively confirm our results given the relatively small number of patients in the studied groups.

## Figures and Tables

**Table 1:** The demographic, anthropometric, clinical, and functional measurements taken during 15-month anti-TNF-α therapy among women with rheumatoid arthritis.

	Before TNFαI Therapy and after Starting TNFαI Therapy			
	T_0_ (Baseline)	T_1_ (3 Months)	T_2_ (9 Months)	T_3_ (15 Months)
	The demographic variables			
Women with RA, *n* (%)	29 (100)			
Premenopausal females, *n* (%)	16 (55.17)			
Postmenopausal females, *n* (%)	13 (44.83)			
Age [years], mean (SD)	46.72 (12.03)			
Disease duration [years], Me (IQR)	5 (3–10)			
RF-positive [*n*], (%)	26 (89.66)			
Anti-CCP-positive [*n*], (%)	24 (82.76)			
	The anthropometric variables			
Growth [cm], mean (SD)	164.03 (6.76)			
Weight [kg], Me (IQR)	64.00 (59.00–70.00)			
BMI [kg/m^2^], Me (IQR)	24.09 (21.09–25.91)			
	The clinical variables			
SJC28 [*n*], Me (IQR)	7 (5–10)	2 (0–3) ^a^	0 (0–0) ^a, b^	0 (0–0) ^a, b, c^
TJC28 [*n*], Me (IQR)	12 (9–16)	4 (2–7) ^a^	1 (0–2) ^a, b^	0 (0–0) ^a, b, c^
VAS [0–100 mm], Me (IQR)	80 (80–80)	40 (30–50) ^a^	20 (10–30) ^a, b^	10 (5–20) ^a, b, c^
DAS28-ESR, Me (IQR)	5.84 (5.68–6.24)	3.92 (3.08–4.42) ^a^	2.75 (2.24–3.13) ^a, b^	2.13 (1.75–2.51) ^a, b, c^
	The disease activity			
Disease activity [*n*], (%)				
High (>5.1)	29 (100)	2 (6.90)	0 (0)	0 (0)
Moderate (>3.2 and ≤5.1)	0 (0)	18 (62.09)	3 (10.34)	0 (0)
Low (≤3.2 and >2.6)	0 (0)	5 (17.24)	13 (44.83)	5 (17.24)
Remission (≤2.6)	0 (0)	4 (13.77)	13 (44.83)	24 (82.76)
	The inflammatory parameters			
ESR [mm/h], Me (IQR)	17.0 (11.0–34.0)	12.0 (9.0–23.0) ^a^	13.0 (9.0–18.0) ^a^	12.0 (8.0–16.0) ^a^
CRP [mg/L], Me (IQR)	6.47 (3.10–14.0)	4.00 (2.0–7.44)	4.0 (2.0–4.3) ^a^	4.0 (1.5–5.0) ^a^
MMP-3 [ng/mL], Me (IQR)	36.69 (19.89–49.97)			16.32 (6.79–19.89) ^a,^*
	The type of TNF-α inhibitor			
TNFαI therapy [*n*], (%)				
Etanercept (Enbrel^®^)	16 (55.17)			
Adalimumab (Humira^®^)	13 (44.83)			

Data are presented as means (SD) or medians (Me), inter-quartile (25th–75th percentile) ranges, or percentages (%). Data were analyzed using one-way RM-ANOVA Friedman’s test. Differences noted for all variables were considered significant at *p* < 0.0083, which was determined by applying Bonferroni correction. Data for MMP-3 were analyzed using Wilcoxon rank-sum test. Differences noted for MMP-3 were considered significant at * *p* < 0.001. Anti-CCP, anti-cyclic citrullinated peptide antibody; BMI, body mass index; CRP, C-reactive protein; DAS28-ESR, 28-joint disease activity score based on erythrocyte sedimentation rate; ESR, erythrocyte sedimentation rate; IQR, inter-quartile range; Me, median; MMP-3, matrix metalloproteinase 3; RA, rheumatoid arthritis; RF, rheumatoid factor; SD, standard deviation; SJC28, swollen joint count out of 28 joints; TJC28, tender joint count out of 28 joints; TNFαI, tumor necrosis factor-α inhibitors; VAS, visual analogue scale. ^a^ statistically significant differences compared to T_0_; ^b^ statistically significant differences compared to T_1_, ^c^ statistically significant differences compared to T_2_.

**Table 2:** The demographic, anthropometric, clinical, and functional measurements taken during 15-month adalimumab or etanercept therapy with respect to women with rheumatoid arthritis.

	Type of TNF-α Inhibitor			
	Etanercept		Adalimumab	
	T_0_ (Baseline)	T_3_ (15 Months)	T_0_ (Baseline)	T_3_ (15 Months)
	The demographic variables			
Women with RA, *n* (%)	16 (55.17)		13 (44.83)	
Premenopausal females, *n* (%)	8 (50)		8 (61.54)	
Postmenopausal females, *n* (%)	8 (50)		5 (38.46)	
Age [years], mean (SD)	44.44 (13.66)		49.54 (9.42)	
Disease duration [years], Me (IQR)	5.00 (3.00–11.00)		3.00 (3.00–9.00)	
RF positive [*n*], (%)	14 (87.50)		12 (92.31)	
Anti-CCP positive [*n*], (%)	13 (81.25)		11 (84.62)	
	The anthropometric variables			
Growth [cm], mean (SD)	164.56 (7.36)		163.38 (6.19)	
Weight [kg], mean (SD) or Me (IQR)	64.50 (58.00–73.80)		64.38 (10.24)	
BMI [kg/m^2^], mean (SD) or Me (IQR)	24.37 (20.69–27.92)		24.07 (3.27)	
	The clinical variables			
SJC28 [*n*], mean (SD) or Me (IQR)	7.31 (4.09)	0.13 (0.34) ^a,^**	6.00 (6.00–10.00)	0 (0–0) ^a,^*
TJC28 [*n*], mean (SD)	14.00 (5.98)	0.19 (0.40) ^a,^**	11.38 (3.55)	0.46 (1.13) ^a,^**
VAS [0–100 mm], mean (SD) or Me (IQR)	80.00 (77.50–80.00)	10.00 (2.50–20.00) ^a,^**	78.85 (11.02)	14.62 (12.16) ^a,^**
DAS28-ESR, mean (SD)	6.00 (0.48)	1.99 (0.68) ^a,^**	5.85 (0.53)	2.09 (0.58) ^a,^**
	The disease activity			
Disease activity [*n*], (%)				
High (>5.1)	16 (100)	0 (0)	13 (100)	0 (0)
Moderate (>3.2 and ≤5.1)	0 (0)	0 (0)	0 (0)	0 (0)
Low (≤3.2 and >2.6)	0 (0)	3 (18.75)	0 (0)	2 (15.38)
Remission (≤2.6)	0 (0)	13 (81.25)	0 (0)	11 (84.62)
	The inflammatory parameters			
ESR [mm/h], Me (IQR)	19.00 (8.00–40.00)	12.50 (6.00–16.50) ^a,^*	17.00 (12.00–32.00)	12.00 (9.00–16.00) ^a,^*
CRP [mg/L], Me (IQR)	6.39 (3.29–10.15)	4.00 (1.95–5.50) ^t^	9.80 (3.10–20.00)	2.00 (1.20–4.10) ^a,^*
MMP-3 [ng/mL], mean (SD) or Me (IQR)	36.07 (15.62–51.62)	14.93 (6.09–20.18) ^a,^**	35.81 (20.34)	17.22 (10.63) ^a,^**

Data are presented as means (SD) or medians (Me), inter-quartile (25th–75th percentile) ranges, or percentages (%). Data were analyzed using paired Student’s *t* test or Wilcoxon’s rank-sum test. Differences noted for all variables were considered significant at * *p* < 0.01 and ** *p* < 0.001. Anti-CCP, anti-cyclic citrullinated peptide antibody; BMI, body mass index; CRP, C-reactive protein; DAS28-ESR, 28-joint disease activity score based on erythrocyte sedimentation rate; ESR, erythrocyte sedimentation rate; IQR, inter-quartile range; Me, median; MMP-3, matrix metalloproteinase 3; RA, rheumatoid arthritis; RF, rheumatoid factor; SD, standard deviation; SJC28, swollen joint count out of 28 joints; TJC28, tender joint count out of 28 joints; TNFαI, tumor necrosis factor-α inhibitors; VAS, visual analogue scale. ^a^ statistically significant differences compared to T_0_ (baseline). ^t^ trend (*p* = 0.064).

**Table 3:** Relationships between circulating levels of cartilage remodeling markers (C2C, PIICP, and COMP) and demographic parameters as well as clinical and laboratory indicators of disease activity in relation to female RA patients at baseline and after 15 months of anti-TNF-α therapy.

Parameter	RA Patients (*n* = 29)			
	T_0_ (Before TNFαI Therapy)			
	C2C [ng/mL]	PIICP [ng/mL]	C2C/PIICP	COMP [ng/mL]
AGE [years]	r = 0.115; *p* = 0.552	r = −0.026; *p* = 0.895	r = 0.071; *p* = 0.714	r = 0.391; *p* = 0.036
Disease duration [years]	r = 0.058; *p* = 0.765	r = 0.063; *p* = 0.745	r = −0.062; *p* = 0.748	r = 0.134; *p* = 0.487
CRP [mg/L]	r = 0.169; *p* = 0.382	r = 0.142; *p* = 0.463	r = −0.126; *p* = 0.514	r = 0.041; *p* = 0.834
ESR [mm/h]	r = −0.025; *p* = 0.899	r = −0.001; *p* = 0.996	r = −0.020; *p* = 0.917	r = −0.066; *p* = 0.735
MMP-3 [ng/mL]	r = 0.082; *p* = 0.673	r = 0.017; *p* = 0.931	r = 0.100; *p* = 0.620	r= 0.083; *p* = 0.670
DAS28-ESR	r = 0.104; *p* = 0.592	r = −0.042; *p* = 0.827	r = 0.061; *p* = 0.755	r = 0.418; *p* = 0.024
SJC28 [*n*]	r = −0.270; *p* = 0.157	r = 0.032; *p* = 0.870	r = −0.191; *p* = 0.320	r = 0.364; *p* = 0.052
TJC28 [*n*]	r = 0.149; *p* = 0.440	r = −0.023; *p* = 0.906	r = 0.050; *p* = 0.800	r = 0.167; *p* = 0.387
VAS [0–100 mm]	r = −0.103; *p* = 0.595	r = −0.135; *p* = 0.486	r = 0.094; *p* = 0.626	r = 0.101; *p* = 0.601
	**T_3_ (15 Months After Starting TNFαI Therapy)**			
CRP [mg/L]	r = 0.071; *p* = 0.714	r = −0.157; *p* = 0.417	r = 0.136; *p* = 0.483	r = −0.131; *p* = 0.500
ESR [mm/h]	r = −0.083; *p* = 0.669	r = 0.013; *p* = 0.947	r = 0.007; *p* = 0.971	r = −0.107; *p* = 0.580
MMP-3 [ng/mL]	r = −0.051; *p* = 0.792	r = 0.066; *p* = 0.733	r = −0.072; *p* = 0.711	r = 0.020; *p* = 0.917
DAS28-ESR	r = 0.140; *p* = 0.469	r = −0.015; *p* = 0.938	r = 0.154; *p* = 0.425	r = 0.081; *p* = 0.675
SJC28 [*n*]	r = 0.189; *p* = 0.325	r = 0.284; *p* = 0.135	r = −0.189; *p* = 0.325	r = 0.162; *p* = 0.400
TJC28 [*n*]	r = 0.220; *p* = 0.251	r = 0.001; *p* = 0.997	r = 0.209; *p* = 0.277	r = 0.143; *p* = 0.461
VAS [0–100 mm]	r = 0.171; *p* = 0.375	r = −0.098; *p* = 0.615	r = 0.142; *p* = 0.463	r = 0.162; *p* = 0.400

Data are expressed as r values (correlation coefficient) determined according to Spearman’s rank correlation. Correlations were considered significant at *p* < 0.05. CRP, C-reactive protein; COMP, cartilage oligomeric matrix protein; DAS28-ESR, 28-joint disease activity score based on erythrocyte sedimentation rate; C2C, collagen type-II C-terminal cleavage neoepitope; ESR, erythrocyte sedimentation rate; MMP-3, matrix metalloproteinase-3; PIICP, procollagen type II C-terminal propeptide; RA, rheumatoid arthritis; SJC28, swollen joint count out of 28 joints; TJC28, tender joint count out of 28 joints; TNFαI, tumor necrosis factor-α inhibitors.

**Table 4:** Multiple linear regression analysis for predictors of serum COMP levels in female RA patients at baseline.

Parameter	COMP in RA Patients (*n* = 29) at Baseline (T_0_)	
	β	*p*-Value
AGE [years]	8.01	0.101
DAS28-ESR	287.74	**0.022**
SJC28 [*n*]	7.44	0.661

Bold values were determined to be significant at *p* < 0.05. COMP, cartilage oligomeric matrix protein; DAS28-ESR, 28-joint disease activity score based on erythrocyte sedimentation rate; ESR, erythrocyte sedimentation rate; RA, rheumatoid arthritis; SJC28, swollen joint count out of 28 joints.

## Data Availability

Data are contained within the article.
